# Gut Health Responses to Nutritional Interventions in Paediatric Crohn’s Disease, Including the Potential Outcomes of Mucosal Barrier Preservation: A Systematic Review

**DOI:** 10.3390/nu18071146

**Published:** 2026-04-02

**Authors:** Ervine Chastine Marind, Fiona McCullough

**Affiliations:** School of Bioscience, University of Nottingham, Nottingham NG7 2RD, UK; chastinervine@gmail.com

**Keywords:** paediatric Crohn’s disease, mucosal barrier, nutrition intervention, diet treatment, intestinal integrity, intestinal permeability, gut barrier, mucosal barrier

## Abstract

**Background/Objectives**: Dietary treatment may play a complimentary part alongside established medical treatment pathways for children with Crohn’s disease. The aim of this review was to explore the impact of a range of dietary treatments, including the capability of preserving the mucosal barrier, during the maintenance phase of Crohn’s disease. **Methods**: Randomised controlled trials and cohort studies were retrieved from five databases (Cochrane library, MEDLINE, EMBASE, ScienceDirect, and Web of Science) and through hand searching (last search: June 2025). In the inclusion criteria, this review only included studies that directly assessed children with Crohn’s disease who achieved clinical remission after the induced phase but simultaneously appeared to have signs of inflammation. **Results**: Six studies were identified, three of which reported outcomes directly associated with the mucosal barrier, while the other studies reported intestinal inflammation and nutritional status. A range of dietary approaches were investigated, with mixed outcomes. A carbohydrate-based diet had a mixed-effect influence on the mucosal barrier, whereas an exclusion diet significantly reduced intestinal inflammation (*p* = 0.01). One study reported that bovine colostrum (BC) milk (a novel approach) demonstrated mucosal integrity improvement, while the timing of partial enteral nutrition (PEN) also improved nutritional status. Importantly, compliance with all these strict regimes is complex and difficult to implement, even with the support of a dietitian. **Conclusions**: Consideration of the most appropriate dietary approach within CD management including remission has reported mixed effects to date. Further research is needed, especially to establish the benefits and any negative consequences of dietary intervention more clearly, and especially regarding mucosal integrity.

## 1. Introduction

Crohn’s disease (CD) is a type of inflammatory bowel disease (IBD) that infiltrates all layers of the intestinal wall [1]. Inflammation can progressively affect any part of the gastrointestinal tract (mouth to anus) [2]. Typically, the standard care to induce remission in children and young people with Crohn’s disease involves conventional glucocorticoids as monotherapy or considers enteral nutrition as an alternative to drug treatment [3]. Although the main concern of drug therapy is to consider the growth development of children [3], these medications do not directly address broader aspects of gut health, including intestinal barrier-related processes.

The management of children with CD is more complex for a range of reasons, including the added complication of more extensive perianal involvement when compared with adults with CD [4,5]. Consequently, children with CD have been exposed to greater medication use and have been reported to exhibit worse outcomes, such as growth impairment [4,5]. Moreover, children with CD frequently exhibit a reduction in the gut microbiome bacteria (Bacteroidetes and Firmicutes), which may associated with the disruption of the epithelial intestinal barrier [6,7]. The disturbance in intestinal epithelial cells may further explain the ongoing gastrointestinal symptoms and the onset of disease flares, even during periods of clinical remission [8]. Given the importance of enhancing intestinal barrier functions in paediatric CD, there is a need to explore the potential of a range of adjunctive strategies that may complement pharmacological therapy by targeting the gut environment, alongside other changes.

Some pharmacological strategies have been proposed to improve intestinal barrier function in celiac disease; however, the evidence regarding IBD remains limited [8]. Several nutritional interventions, including the provision of dietary fibre and the prohibition of high fat intake, have been reported to exert potential beneficial effects on intestinal barrier maintenance through modulation of the gut environment [8,9,10]. Consumption of bovine colostrum milk has been proposed as an alternative approach to IBD due to its potential to block inflammatory signalling in intestinal epithelial cells in animal studies [11]. However, the extent to which diet-induced alterations in the gut environment contribute to a tight mucosal barrier or further conversely influence the risk of disease relapse remains unknown.

For remission induction, exclusive enteral nutrition (EEN) for 6–8 weeks has been widely recommended as a first-line treatment for children according to consensus guidelines conducted by ECCO/ESPGHAN [12]. Upon completion of EEN, the occurrence of mucosal healing is shown [13] due to its composition that alleviates mechanical stress within the wall of the intestine [14]. However, the ability of EEN to sustain remission is limited [15] and, thus, the incidence of relapse post-EEN has been common [16,17]. Following this, well-established mucosal barriers are prone to rebound. Therefore, children with CD who achieved remission during induction may not have had intestinal barrier function restored.

The only included trial that reported improvement in intestinal permeability function through a consistent decreased L/R ratio was the bovine colostrum milk supplementation trial. This improvement is biologically plausible due to bioactive compounds within bovine colostrum that directly increase tight junction (TJ) proteins in the intestinal epithelium [18] (Table A4).

The effect of bovine colostrum (BC) on the intestinal epithelial barrier can be demonstrated through two mechanistically distinct but complementary pathways (Table A4). In the direct pathway, BC regulates the expression of tight junction proteins due to the existing growth factors (EGF, TGF-β, and IGF-1) [19]. Then, the increased TJ proteins (occludin and claudin-3) enhance mucin secretion by goblet cells and reduce the pathogen around the intestinal barrier. Thus, it directly restores intestinal barrier integrity, reflected by a reduction in the lactulose/rhamnose (L/R) ratio, as demonstrated by the included trial in this review.

These uncertainties highlight various gaps in understanding, including how nutritional interventions shape gut health responses during the maintenance phase of paediatric Crohn’s disease and whether preservation of the mucosal barrier represents a potential outcome upon these approaches. Therefore, this review aims to examine gut health responses to a range of dietary interventions during the maintenance phase of paediatric Crohn’s disease in order to identify current knowledge gaps to help guide future research.

Gut health response within this review has included any disease activity, inflammation marker and possibility of gut microbiota shift activity following diet intervention that may affect the intestinal mucus layer. These domains may collectively reflect the functional state of the intestinal barrier function.

The intestinal epithelial barrier is not a passive wall but an active multi-layered defense system. It consists of three distinct but interdependent components, such as tight junctions (TJs) for maintaining paracellular permeability and barrier integrity, goblet cells with their mucin production to secrete a protective mucus layer, and antimicrobial peptides (AMPs) for immune response [20,21]. In Crohn’s disease, the dysfunction of all three components in the intestinal epithelial barrier has been recognised as a possible contributor to the disease pathogenesis [22].

Given the role of the intestinal epithelial barrier in maintaining gut homeostasis, its functional metrics can be assessed through direct and indirect measures. The direct assessment for intestinal permeability in humans is most commonly achieved through quantifying the movement of large and small size molecules, such as lactulose and rhamnose (L/R ratio), across the epithelium [23]. However, since direct permeability alone does not capture the full clinical consequences of barrier failure, indirect measures are also additionally employed across three domains, such as mucosal inflammation, nutritional outcome, and disease activity.

The assessment of mucosal inflammation is most commonly achieved through faecal calprotectin (FC) levels, which serve as surrogate markers for intestinal inflammatory activity [24]. Nutritional outcomes, including body weight and micronutrient levels, reflect the malabsorption consequences of barrier dysfunction. Disease activity is quantified using validated assessments such as the Paediatric Crohn’s Disease Activity Index (PCDAI) in children. These assessment tools allow a more comprehensive look toward gut health responses in paediatric Crohn’s disease following a provision of diet.

## 2. Materials and Methods

This systematic review was conducted based on the preferred reporting items for systematic reviews and meta-analyses (PRISMA) checklist [25]. This review included full-text randomised trials, open-label studies, and prospective or retrospective cohort studies investigating dietary treatment in paediatric Crohn’s disease during post-induction remission.

Clinical remission was defined by the Paediatric Crohn’s Disease Activity Index (PCDAI) or Weighted Paediatric Crohn’s Disease Activity Index (wPCDAI) score < 10 and <12.5 points, respectively [26]. To represent high risks of exacerbations, studies involving children with elevated faecal calprotectin (FC) levels > 250 µg/g were considered eligible [27]. Therefore, studies whose primary endpoint was only to achieve an induction of remission were excluded. Drug therapy remains essential for sustaining remission [12], thus studies combining drug and dietary treatment were eligible. Studies only focusing on medical intervention were excluded.

Table 1 summarises the detailed inclusion and exclusion criteria based on the PICO framework.

The primary outcome for this review was the intestinal barrier’s capacity to restore or maintain the tight junctions. The term “preservation of mucosal barrier” indicates normal permeability and an absence of inflammation [23]. The intended outcome of this review was examined through non-invasive tests (lactulose/L-rhamnose) for evaluating intestinal barrier function [28] and endoscopic measures (SES-CD or Lewis score) [29,30]. Studies lacking emphasis on the mucosal barrier were excluded.

**Figure 1:**
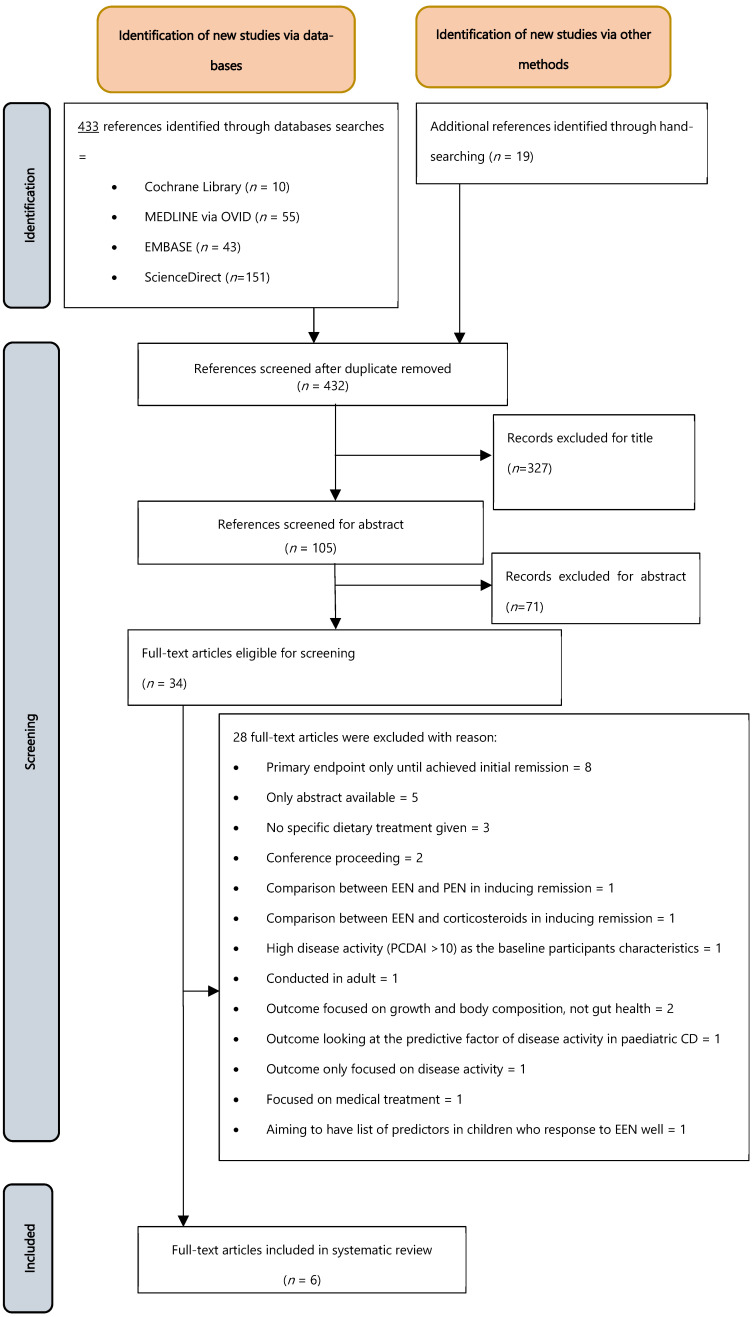
Flow chart of selection studies.

Figure 1 summarises the study selection process. An extensive search was performed in five databases and through hand searching until June 2025, mainly using references from www.nutritionaltherapyforibd.org. No publication time restriction was applied; however, limitations of the full-text articles, human studies, and English languages were included at the screening stage. Thirty-four full-text articles were screened against inclusion and exclusion criteria. Twenty-eight articles were excluded, mostly due to irrelevant outcomes, leaving six studies for final analysis. The full search descriptions employed for each search engine are presented in Table A1.

**Figure A1:**
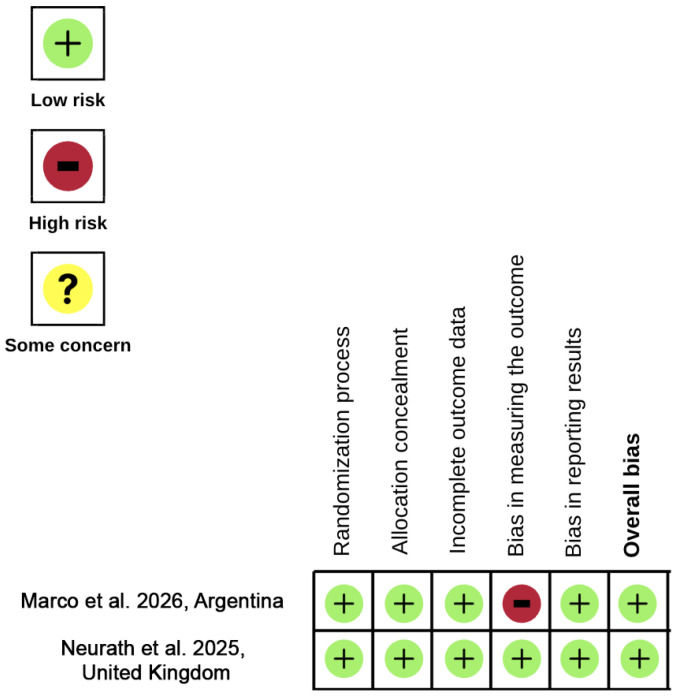
RoB 2 for measuring risk of bias in randomised trials [21,22].

**Figure A2:**
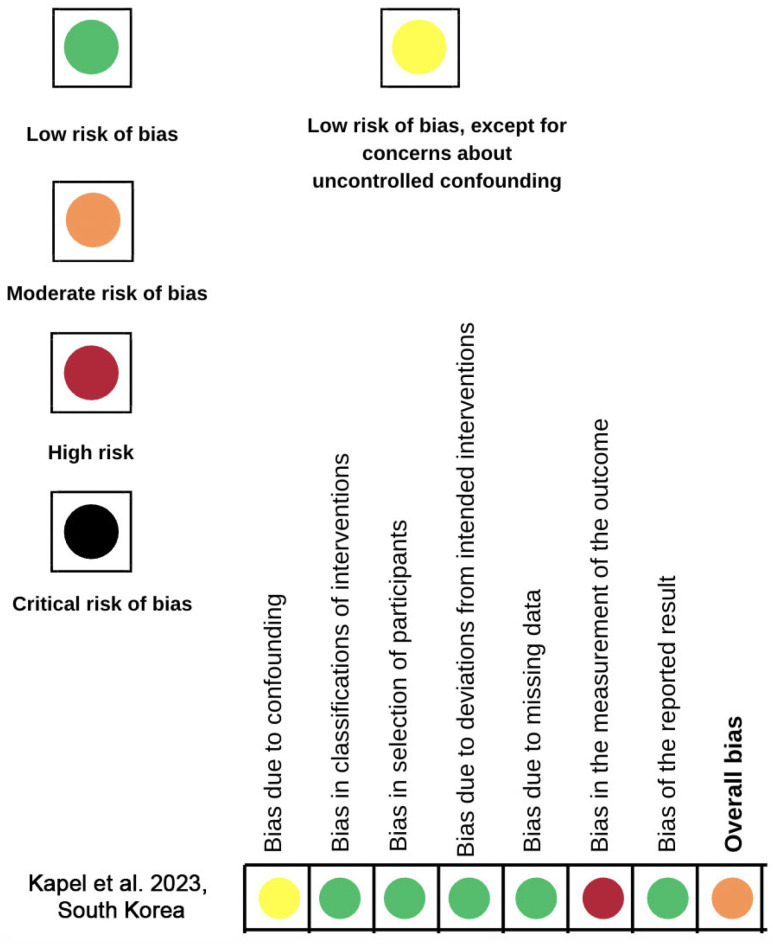
ROBINS-I for non-randomised trials [24].

Each study was appraised for its risk of bias according to the study design (Appendix A). Three cohort studies were evaluated using the Newcastle–Ottawa Scale (NOS) (Table A2). Moreover, two randomised trials conducted in [31,32] were assessed using the RoB 2 tool [33] (Figure A1). Lastly, the only non-randomised trial [34] was examined with the ROBINS-I tool [35] (Figure A2).

## 3. Results

### 3.1. Characteristics of the Studies

Of the six eligible studies, five were conducted during the maintenance phase of CD, and one study [36] included the induced remission phase. Participants were children in induced remission but with a high risk of relapse. Most studies combined dietary and pharmacological therapy during the maintenance phase, except [37], which evaluated diet as the sole treatment (Table 2). Overall, all studies showed a low risk of bias, with the exception of one study [34] that demonstrated moderate risk; however, due to the low number of studies, the decision was made not to exclude it (Figure A2).

### 3.2. Clinical Outcomes Relating to Gut Health Responses in Crohn’s Disease

Various clinical outcomes interpreting gut integrity are explained in Table 3.

#### 3.2.1. Lactulose Rhamnose (L/R) Ratio

Only one study [32] assessed the mucosal barrier using the lactulose-rhamnose (L/R) ratio; however, it lacked a defined normal cut-off value. Thus, the results were interpreted based on trends. The treatment group displayed a consistent decline in the L/R ratio from baseline to week 6 (0.030 to 0.0046, *p* = 1.0) and further at week 12 (0.0021). Similarly, the placebo group that started on bovine colostrum (BC) at week 7 also showed a reduction in the L/R ratio at week 12 but demonstrated a greater ratio than the treatment group (0.0033 vs. 0.0021, respectively). Children who received bovine colostrum since week 1 were reported to have a lower final L/R ratio (0.0021).

#### 3.2.2. Simple Endoscopic Score (SES-CD) and Lewis Score

Two studies employed SES-CD and Lewis score to investigate inflammation in the colon and small bowel, respectively [36,37]. Both scores reported visible ulcers through ileocolonoscopy (SES-CD) or small intestine lesions through capsule endoscopy (Lewis score). Moreover, both of the scores were used to evaluate mucosal healing. The key difference lies in the cut-off definition of complete mucosal healing. SES-CD defines complete healing as a score of zero (no ulceration), while the Lewis score considers a value below 135 to indicate normal mucosa.

Overall, the findings from [37] whose endoscpic outcomes defined by SES-CD resulted in a range from mucosal healing to worsened state. However, it was demonstrated that only one child appeared to have complete mucosal healing (SES-CD from 20 to 0) at 12 weeks post-diet treatment.

On the other hand, a study that used the Lewis score [36] reported an initial decline from week 0 until week 12 (2153 ± 732 to 960 ± 432, *p* = 0.012), followed by a rebound average of 1046 ± 372 (*p* = 0.091) at the 52-week follow-up. During the week 12 assessment, four children showed an LS score < 135. However, until week 52, normal mucosa was only observed in three patients, with two patients who had mucosal healing at week 12 and surprisingly one patient who did not achieve mucosal healing (LS: 143) following 12 weeks of treatment.

#### 3.2.3. Faecal Calprotectin (FC)

The measurement of intestinal inflammation was depicted in a form of faecal calprotectin (FC) in four studies. The cut-off normal value for FC concentration is established at 250 μg/g, with the exception of two studies [32,37] that employed the normal value at 50 μg/g. Out of four studies, only one study [37] had a finding of an elevated FC (>50 μg/g). To the contrary, the other three studies identified reduced intestinal inflammation as measured by an FC level lower than their determined cut-off values.

#### 3.2.4. Paediatric Crohn’s Disease Activity Index (PCDAI)

The subjective scale to measure disease activity known as PCDAI was measured in all studies, with the exception of one study [37]. Overall, children who received the treatment intervention demonstrated a declining trend in their PCDAI scores [31,34,36,38]. However, in one study [38], a significant reduction in PCDAI scores was only reported among children who did not achieve clinical remission at baseline of intervention (PCDAI ≥ 10), with scores dropping from 20 to 5 at week 12, *p* = 0.0002. Although another study [32] reported a median change in PCDAI scores, the interpretation of these findings remains uncertain due to the limited clarity in the presentation of results.

#### 3.2.5. Body Mass Index (BMI) and Nutrient Absorption

The use of BMI as an outcome to reflect nutritional status was found in one RCT and one prospective pilot study [31,36]. Both studies presented a gain and loss of BMI z-score in children following the intervention. Meanwhile, the gut ability to reflect nutrient absorption was well-documented in one study [34]. The treatment group was found to have higher status of calcium, magnesium, phosporus, and vitamin A, although the differences were not statistically significant.

### 3.3. Nutritional Interventions in the Maintenance Phase of Paediatric Crohn’s Disease

All the included six studies investigated five different dietary treatments in their attempts to preserve the mucosal integrity for paediatric CD with a high risk of relapse. Detailed treatments are summarised in Table 4. The results of each diet treatment are presented in Table 5.

#### 3.3.1. Crohn’s Disease Exclusion Diet (CDED) and Partial Enteral Nutrition (PEN)

CDED + PEN were studied in one randomised controlled trial [31] and one cohort study [38]. Both studies employed CDED with an additional PEN approach in children who had already achieved clinical remission (PCDAI < 10) but who were at high-risk of relapse, as indicated by an elevated FC concentration (>250 µg/g). The results demonstrated a dropped FC level to below 250 µg/g following the provision of a CDED + PEN diet. However, a significantly reduced FC level was profoundly found in children with clinical remission at baseline (Table 5) [38].

The major reason for CDED + PEN discontinuation was due to the lack of therapy efficacy [38]. On the other hand, the provision of CDED + PEN during the maintenance phase also found to induced clinical relapse (*p* = 0.149) and intensification of biological treatment in one child (*p* = 0.005) [31]. However, at study conclusion, the provision of CDED + PEN during the maintenance phase was beneficial in reducing the inflammation burden, as evidenced by decreased FC levels in both studies and less need for drug treatment escalation [31].

#### 3.3.2. Specific Carbohydrate Diet and Modified Specific Carbohydrate Diet

The specific carbohydrate diet [36] and its modifications [37] for children with Crohn’s disease were investigated in two studies. Both studies shared a similar focus in their research aims: to serve as an evaluation towards mucosal healing following treatment (Table 5).

The principle of the specific carbohydrate diet (SCD) prioritises fresh food and prohibits instant food products [36]. Children with Crohn’s disease in the study were allowed to consume vegetables, fruit, legumes, and home-made yoghurt with no additional sugar. Meanwhile food that must be taken into avoidance were canned foods, all grains, particular beans, dairy products, and instant products containing refined sugar. A key finding from this treatment was that most of the participants increased their overall food consumption by the end of week 12. The acceptability of the diet was observed after 3 months of treatment; however, only 7 out of 9 patients chose to continue SCD (Table 4).

On the other hand, the instruction of a modified specific carbohydrate diet (mSCD) allowed re-exposure to the limited foods in SCD, such as rice, oat, potato, and quinoa (Table 4) [37]. The effect of this approach was demonstrated through changes in the upper gastrointestinal tract and ileal disease phenotype, as shown in Table 5. Interestingly, colonic inflammation was shown in children who showed improvement after the administration of mSCD, although their inflammation activity was low.

Both studies’ conclusions revealed that the provision of the specific carbohydrate diet and its modification resulted in a lack of complete mucosal healing. The lack of mucosal healing was reflected by various endoscopic results measured by SES-CD and increased FC concentration (>50 μg/g) in 71% children, with the median score shown in Table 5 [37]. However, it is worth noting that their study limitations were due to the small sample size (n = 7) and depended on hospital reports [37].

Similarly, mucosal changes were also less consistent in children who received the SCD intervention [36]. However, the study found a profound clinical improvement by decreased PCDAI through the entire 52 weeks (Table 5).

#### 3.3.3. Bovine Colostrum (BC) with General Diet

A randomised controlled trial challenged the antimicrobial and immunomodulatory content in bovine colostrum (BC) to identify its effect on intestinal integrity, inflammation and quality of life in paediatric Crohn’s disease [32]. In a 12-week intervention, the trial conducted a blinded random allocation with the formula mentioned in Table 4. In the first blinded phase, 12 children were administered BC, with the dose adjusted according to their tolerability. Subsequently, 12 children who received BC continued to have BC in an open phase until week 12. In contrast, 11 children in the control group had to take the placebo milk during the first 6 weeks prior to being instructed to drink BC in an open phase.

The total percentage of children who reported feeling better after consuming BC was higher in the placebo group (85.7% vs. 71.4%, *p* = 0.56). However, withdrawal rates during the open phase were common in the placebo group compared with children who previously had BC (44.4% vs. 12.5%, *p* = 0.29). The reason of higher dropout onset in the placebo group was due to the low questionnaire return rate for weeks 7–12. However, symptoms associated with side effects after the consumption of BC and placebo milk were similar, with children commonly having wind and lacking energy (Table 5).

During 12 weeks of intervention, both the treatment and the control group were allowed to consume a general diet and continued to have biologic therapy. The findings indicate that the provision of BC did not significantly reduce intestinal inflammation, as the median decrease in FC levels was higher in the placebo group (−9 drop in BC vs. −45.5 in placebo, *p* = 0.59). However, the mucosal barrier integrity in children receiving BC was demonstrated to have a lower L/R ratio than the placebo group (0.0021 vs. 0.0033). Therefore, the study concluded that the small number of participants (n = 23), infliximab consumption and short treatment duration possibly limited the potential in revealing the efficacy of BC for improving clinical and laboratory outcomes [32].

#### 3.3.4. Short-Term Partial Enteral Nutrition (PEN) with General Diet

The investigation of short-term PEN (SPEN) as an adjuvant therapy to a general diet during the maintenance phase was conducted for four weeks [34]. In the study, children with severe Crohn’s disease who had already achieved initial remission were instructed to consume partial enteral nutrition (PEN) to measure its effect on micronutrient status, body weight, and disease activity. The standard polymeric formula given in this open-label study is detailed in Table 4. However, the details of the general diet consumed were not clearly defined.

The findings revealed that children in the SPEN group had significantly improved nutrient absorption and disease severity until the follow-up assessment at week 52 (Table 5). Although the findings were profoundly promising, the study concluded that the long follow-up interval was one of the limitations, along with the small number of participants (n = 34) and the study design.

## 4. Discussion

This systematic review aimed to explore gut health responses, including the potential outcomes related to mucosal barrier preservation, following a range of nutritional interventions during the maintenance phase of paediatric Crohn’s disease. The number of papers and subjects was low overall, although the gut health response outcomes reported included disease activity measured as PCDAI [31,32,34,36,38] and reported as significant inactive or mild symptoms in children with Crohn’s disease after diet intervention. Importantly, the data available relating to direct gut health outcomes, measuring intestinal barrier function, were extremely limited, with only one study evaluating intestinal permeability using the lactulose/rhamnose (L/R) ratio [32]. The study reported a significant lower L/R ratio, suggestive of improved intestinal barrier function.

To our knowledge, this is the first systematic review focused on gut health responses to establish whether it was possible to suggest overall effectiveness of nutrition interventions for paediatric CD based on the preservation of intestinal integrity during the maintenance phase. Since the constant inflammation in paediatric CD affects BMI and nutritional status, these outcomes were considered also relevant.

### 4.1. Direct Measurement of Intestinal Permeability

#### Bovine Colostrum Intervention

This systematic review identified one study using bovine colostrum (BC) milk [32] as a sole dietary intervention that directly assessed intestinal permeability using the lactose/rhamnose (L/R) ratio. The included trial reported an improvement in intestinal permeability function following BC milk supplementation through a consistent decreased L/R ratio. This improvement is biologically plausible due to bioactive compounds within bovine colostrum that directly increase tight junction (TJ) proteins in the intestinal epithelium [18] (Table A4).

Meanwhile, through an indirect pathway, antimicrobial peptides in bovine colostrum, including lactoferrin, immunoglobulin (IgG), and lysozyme, reduce bacterial translocation across the epithelial surface, which attenuates chronic mucosal inflammation that induces barrier dysfunction [39]. As a result of this reduced inflammatory burden in the intestinal epithelium, there is improved mucosal absorption capacity, reflected through increased BMI. However, in the included trial, both groups received BC and the placebo group experienced small weight gains. Therefore, it was not possible to directly attribute to the BC milk itself. The other underlying reason might be due to the normal growth of the children over time, since BC supplementation was consumed alongside with the normal diet.

The methodological quality of the bovine colostrum study in this review was assessed using the Cochrane risk of bias (RoB 2) tool, with an overall judgement of low risk of bias across the evaluated domains (Figure A1). Notably, the L/R ratio was measured using a standardised and validated protocol, supporting the intestinal permeability outcome. However, reduction in the L/R ratio that was observed between groups following BC supplementation did not reach statistical significance. This finding may reflect a small sample size obtained in the included trial. In this regard, the current evidence base is insufficient to draw conclusions regarding the efficacy of bovine colostrum in improving intestinal permeability in paediatric Crohn’s disease.

For practical consideration, the method of bovine colostrum supplementation raises questions due to the addition of flavouring agents to enhance palatability. While this approach is understandable in order to obtain acceptance from children, emulsifiers that exist in the flavouring agents have been implicated as a contributing factor to the exacerbation of IBD [40].

### 4.2. Indirect Outcomes of Mucosal Inflammation

#### 4.2.1. Crohn’s Disease Exclusion Diet (CDED) and Partial Enteral Nutrition (PEN)

Both studies employed the Crohn’s disease exclusion diet and partial enteral nutrition (CDED + PEN) [31,38], reporting a tendency toward a reduction in faecal calprotectin (FC) levels, suggesting a consistent decrease in mucosal inflammation across different study designs (Table 4). However, both findings warrant careful interpretation. In the Argentinian study [31], the final FC level was still above the clinical meaningful threshold of 250 µg/g as suggested by ECCO-ESPGHAN guidelines [41], with the level decreased from 750 to 339 µg/g (*p* = 0.0048) following 12 weeks of CDED + PEN intervention. Meanwhile in the Polish study [38], an FC level < 250 µg/g at week 12 was only achieved in children who reached remission at baseline. This indicates that, while CDED + PEN generated a consistent reduction in mucosal inflammation, the 12-week period intervention appeared insufficient to achieve the complete absence of mucosal inflammation in paediatric Crohn’s disease.

The biological basis for the FC level reduction after CDED + PEN can be explained through the microbiome remodelling rather than the simple SCFA-mediated pathway. The CDED regime is specifically designed to exclude dietary components that promote intestinal dysbiosis and intestinal barrier impairment, such as animal fat and emulsifiers [42]. At the same time, it allows mandatory resistant starch and fermentable fibre as substrates that can produce short-chain fatty acids (SCFAs). Evidence from another randomised study [43] demonstrates that CDED + PEN progressively decreased the abundance of Proteobacteria towards levels observed in healthy controls. Critically, despite an upregulation in SCFA synthesis pathways and an increase in SCFA-producing bacteria (Firmicutes) after CDED + PEN intervention, remission was not associated with increased faecal SCFA concentrations [43]. This finding is important because it challenges the assumption that SCFA produced by dietary fibre within a CDED regime is the primary driver for intestinal mucus production and maintenance of the structural intestinal mucosal barrier [44]. However, a complete correction of bacteria similar to a healthy population was not achieved within 12 weeks of CDED + PEN intervention [43]. It may explain why a substantial proportion of patients in both the Argentinian and Polish studies failed to achieve complete FC level normalisation within the study period.

Despite the biologically plausible mechanism of CDED + PEN toward gut health response and low risk of bias quality assessment, it remains premature to conclude that CDED + PEN can be employed as a maintenance dietary therapy to sustain remission in paediatric Crohn’s disease. The findings in the included trial [31,38] suggest that adherence to CDED + PEN is achievable in children and adolescents but still requires sustained professional support. This regime is meticulous in its requirements, encompassing mandatory food categories, strict prohibited items, and phase-specific formula proportions that demand collaboration between children, caregivers, and trained dietitians. This level of support may not be evenly accessible across healthcare settings.

#### 4.2.2. Short-Term Partial Enteral Nutrition (PEN)

The included study examined short-term partial enteral nutrition (short-term PEN) [34] as a 4-week adjunct to medical induction therapy in children with severe Crohn’s disease. The findings showed a significant improvement in nutritional status and body weight in the SPEN group compared with non-SPEN over one year of treatment (*p* < 0.001). However, it is important to note that the primary outcome of the included study [34] was nutritional status recovery, not the absence of mucosal inflammation or barrier restoration (Table 5). Therefore, it becomes critical when interpreting the findings across the enteral nutrition literature.

The improved nutrition status after short-term PEN is biologically plausible. The polymeric formula within short-term PEN attenuates intestinal inflammation through direct anti-inflammatory effects on enterocytes, partially independent of microbiome remodelling [45]. This may create less harm in the mucosal environment where epithelial repair may occur. In children with active CD where transmural inflammation directly reduces absorptive surface areas and impairs mucosal recovery, even short-term formula supplementation may be meaningfully beneficial for nutritional absorption by the restoration of a structural and functional intestinal epithelium. It should be noted, however, that the short-term PEN included in the trial [34] did not directly measure any barrier function. Therefore, the proposed pathway between short-term PEN, mucosal barrier restoration, and improved nutrition absorption remains biologically inferred rather than empirically demonstrated in the study.

In regard to the quality of the study, short-term PEN treatment demonstrated a moderate overall risk of bias based on ROBINS-I assessment (Figure A2). Critically, the allocation of children to the SPEN group versus non-SPEN was not randomised. It was directly decided that children with severe Crohn’s disease received SPEN while those with mild-to-moderate disease received a general diet alone from baseline until 1 year of treatment. This introduces a significant confounding by indication. The improved outcomes showed in the SPEN group are not surprising given that they received additional caloric and protein delivery through formula supplementation on top of their general diet. It is therefore premature to attribute the nutritional improvement as an outcome from short-term PEN treatment. Furthermore, the general diet composition in the non-SPEN group was not recorded, leaving the possibility of pro-inflammatory foods consumed by the participants. Without dietary records, therefore, it is impossible to draw a comparison and fair judgment towards the use of short-term PEN as a maintenance diet for Crohn’s disease.

While partial enteral nutrition (PEN) is an established option for the induction of remission within European CD guidelines [46], the use of enteral nutrition with timing adjustments as a maintenance strategy represents a whole new concept. The recent CD-HOPE trial [47] examines the cyclic exclusive enteral nutrition (EEN) (2 weeks of full EEN across six cycles, interspersed with 6 weeks of unrestricted diet) against daily PEN (covering 25% of total caloric needs) with an unrestricted diet for 52 weeks. The trial underscores the dosing strategy and timing of enteral nutrition as a maintenance diet. The microbiome analysis revealed that cyclic EEN maintained a lower microbial alpha diversity, interpreted as a beneficial bacterial modulation over PEN administration. This finding implies that the concentration and periodicity of enteral nutrition delivery appear to determine the microbiome modulation and likelihood of durability for remission, rather than cumulative formula intake alone. In regard to the review, this means that the short-term PEN [34] and the maintenance PEN described in the CD-HOPE trial [47] should be interpreted as clinically distinct strategies. In addition, the PEN concept of providing 25% of daily calories with an unrestricted diet—the most common studied maintenance diet form in CD—is insufficient as a standalone maintenance strategy for paediatric populations with CD.

In the context of practicality, this dietary regime involving enteral nutrition may carry a risk of reinforcing maladaptive eating behaviours in children who may already associate food with pain, relapse, or loss of control, as children and adolescents with CD have been reported to show food-related reduced quality of life [48]. The concern is not that the formula is harmful but that a child who experiences formula-associated remission may internalise a harmful belief that avoiding normal food might be protective.

#### 4.2.3. Specific Carbohydrate Diet (SCD) and Modification of Specific Carbohydrate Diet (mSCD)

The two studies examined mucosal lesion outcomes following dietary carbohydrate restriction in paediatric Crohn’s disease, reaching different conclusions [36,37]. In the specific carbohydrate diet (SCD) [36], only four of nine children achieved mucosal healing (Lewis score < 135) at week 12, with only three children maintaining normal mucosa at 52 weeks. In the modified specific carbohydrate diet (mSCD) [37], only one of seven children achieved complete ileocolonic mucosal healing (SES-CD = 0) but continued to have persistent colonic chronic inflammation. This finding is the central observation requiring explanation.

Both studies were assessed by overall judgment as low risk of bias in this review (Table A2), reflecting that each study was conducted within the cohort design framework. However, low risk of bias from the studies and the evidence derived are not equivalent judgments. In this review, the certainty of evidence in the dietary restriction of carbohydrate as a strategy for mucosal healing in Crohn’s disease is likely to be low. In the SCD study [36], the enrolled children had actively requested dietary alternatives to pharmacological therapy, which reflected the readiness of motivation, family support, and likely pre-existing dietary awareness. In this case, the selection bias was not merely a concern but an internal validity concern because the observed mucosal improvement may partly reflect the effect of high adherence and motivated self-management rather than the specific carbohydrate composition in SCD.

On the other hand, regarding the modified specific carbohydrate diet (mSCD) [37], the methodology employed may limit the interpretation of the diet intervention itself. First, the inclusion criteria for the retrospective cohort focused on children who had already employed SCD/mSCD for >3 months and had undergone a follow-up endoscopy after the diet. Second, the adherence and definition of mSCD could not be determined as the study design only examined the retrospective data based on parents’ and children’s recall of the diet history. Thus, it may lead to an unmeasured confounder that the prescribed mSCD and the diet consumed by the children may not demonstrably be equivalent. However, this limitation does not weaken the negative mucosal finding in children who consumed mSCD [37]. If the only variable adherence was present in the mSCD study and some children had poor adherence to mSCD (consumed refined sugar or processed carbohydrates), then the diet actually consumed was even less restrictive than mSCD as defined. Yet, even under those conditions, complete mucosal healing of the entire gastrointestinal tract was not demonstrated in any children. Instead, only one child achieved complete ileocolonic healing but remained with chronic upper gastrointestinal inflammation [37]. The logical implication derived is that if a diet less restrictive than mSCD failed to induce mucosal healing, then perfect adherence to mSCD would not be expected to derive a superior result.

The trial conducted by Suskind [49] further explains what can be concluded about both SCD and mSCD under real-world conditions. Although all ten participants achieved clinical remission (PCDAI < 10) at 12 weeks, the provision of the diet regime may limit the external validity of this finding. In that study, SCD and mSCD diets were prepared by a chef throughout the trial, an infrastructure that is not available to patients in clinical practice and fundamentally becomes a distinct feature related to real-world dietary therapy [49]. The 100% clinical remission rate was therefore achieved under conditions of maximal dietary facilitation that cannot be generalised beyond the trial setting. Therefore, clinical remission is achievable upon short-term outcomes of SCD and mSCD under the consistent support of meal preparation.

The mechanistic rationale for dietary carbohydrate restriction in Crohn’s disease on gut health response is based on an indirect pathway. The diet does not deliver anti-inflammatory compounds directly but instead remodelled the gut microbial environment (Table A4), which in turn may influence the intestinal epithelium. The microbiome data from the Suskind study [49] provide the only available evidence that tests the proposed mechanism of SCD, mSCD (adding oats and rice), and whole food diet (eliminating wheat, sugar, corn, milk, and food additives) in paediatric populations who achieved clinical remission. The finding showed that butyrate-producing taxa (*Roseburia hominis*, *Roseburia intestinalis*, *Faecalibacterium prausnitzii*, *Anaerobutyricum hallii*, and *Eubacterium eligens*) increased in abundance in four of five children, while *Escherichia coli* decreased in more than three children. This is consistent with the proposed pathway that the elimination of disaccharides and most complex starches removes the primary fermentable substrate for pathobionts, particularly Escherichia species that are commonly present in active Crohn’s disease [50,51]. However, the authors explicitly note that the microbiome changes were highly patient-specific and taxonomically inconsistent across participants, with within-patient microbiome similarity being greater than between-diet or between-timepoint similarity [49]. Therefore, the mechanism occurred in the predicted direction in most patients but was not a uniform response.

For practicality, both the specific carbohydrate diet (SCD) and the modified specific carbohydrate diet (mSCD) might be biologically plausible but not practically sustainable. The evidence base from the included cohort in the included trial [36,37] has two critical limitations that prevent strong recommendations for a carbohydrate diet as a maintenance diet in Crohn’s disease: (1) mucosal healing was only documented in one child who received mSCD (SES-CD score 0) but maintained inflammation [37] and two children on SCD [36] and (2) the only adult RCT found no advantage to the specific carbohydrate diet over the Mediterranean diet in achieving symptomatic remission [52]. Additionally, the case report from three children revealed that clinical remission following the carbohydrate diet was achieved but failed to show complete mucosal healing [53].

### 4.3. Strengths and Limitations

The main strength was the inclusion of direct and indirect mucosal integrity outcomes. This review applied strict inclusion criteria to assure the baseline characteristics of included participants who had passed the induced remission but had a high risk of inflammation. However, the main weakness was low sample size numbers and the heterogenous endpoints within the small number of papers identified, meaning a meta-analysis could not be conducted. The reported findings should be interpreted with caution, and the novel dietary interventions are unlikely to be suitable for widespread use due to the high level of parental or carer commitments to ensure adherence and would work best with multi-disciplinary team support, which is not always available. All the included studies were from Western populations; hence, caution needs to be applied with interpretation of these results to other populations, e.g., the Asian population.

### 4.4. Future Recommendations

There is a need for further focused research exploring the maintenance phase of CD, particularly investigating a range of outcomes measuring intestinal integrity and indirect markers.

Conducting a multicentre randomised trial would allow the involvement of a larger number of participants. Additionally, a prospective trial would establish the differing effects of diet interventions. Recruiting children from all continents, e.g., Asia and Europe, would allow comparison with previous studies.

Finally, the CDED is present mostly formulated from Western food ingredients. Therefore, investigating this diet with regard to Asian children would likely add new insight on the effect of food composition upon its capacity to preserve the gut barrier. In an era of personalised treatment, formulating more tailored CDED diets would be a helpful development.

## 5. Conclusions

This review has indicated that the evidence base regarding diet and mucosal integrity is extremely limited. Novel interventions require more research before conclusions can be drawn; hence, already established interventions remain the most appropriate and effective way to support maintenance of remission. This approach reports some reduction in intestinal inflammation, while other interventions report more inconsistent outcomes and adverse effects overall. However, for all dietary interventions, the practicality of complying with the diet requires caution and support from health care professionals, including dietitians. Gut health is a topical area with emerging evidence; hence, for children with Crohn’s disease, any novel treatments to support gut integrity and overall quality of life should be explored further in the future

## Figures and Tables

**Table 1:** Inclusion and exclusion criteria.

**Inclusion criteria**	Population	Children and young people (CYP) ≤ 18 years old diagnosed with Crohn’s disease. Have passed the induction of remission phase and are undergoing maintenance phase.
	Intervention	Any form of diet modification or nutrition strategy (PEN, CDED-PEN, CDED, or other food-based intervention, consumed as a drink or a meal. No restriction regarding the amount of energy and macronutrients provided. No restriction regarding the route of nutrition intervention (oral or through nutrition support). Diet intervention conducted after the EEN phase or after the induction of remission achieved.
	Comparison	Unrestricted diet. Standard advice diet along with medical or biological treatment. Placebo.
	Outcomes	Primary outcome: preservation of mucosal or intestinal barrier integrity. Secondary outcomes: BMI and nutritional status.
	Study design	Controlled trials, randomised controlled trials, prospective open-label study, prospective or retrospective cohort studies.
**Exclusion criteria**		Studies measuring the intervention only until the induction of remission. Studies only covering the diversity of microbial changes following dietary treatment. Studies only demonstrating the post-induction remission condition, without specifically investigating the diet intervention. Studies with primary outcome as nutritional status without measuring disease activity and gut health condition. Studies with subject characteristics not in remission, indicated by a PCDAI score > 10. Case reports, case series. Conference proceeding without full-text document. Cross-sectional studies.

**Table 2:** Characteristics of the included studies.

Author, Country, Length of the Intervention	Study Design	Age ^1^ (Years)	Subjects (*n*)	Gender	Disease Status of Participants	Drug Treatment	Type of Diet Treatment, Comparator	Phase
Argentina, 12 weeks [31]	Open label randomised controlled trial	10.65 and 13.59	21	M (45.5%), F (54.5%)	Children in clinical remission (PCDAI * < 10), but have FC ** > 250 µg/g)	Mesalamine, Prednisone, Infliximab, Adalimunab, Vedolizumab	CDED + PEN, regular diet	Maintenance
United Kingdom, 12 weeks [32]	Randomised controlled	15.4 and 14.9	23	M (61%), F (39%)	Either had remission or mild disease	Infliximab	Bovine colostrum milk and continue usual diet, placebo milk and continue usual diet	Maintenance
South Korea, 1 year [34]	Prospective open label study	14 and 13.8	34	M (50%) and F (50%)	Children with severe degree of Crohn’s disease (PCDAI > 45) either with first diagnosis or relapse condition during maintenance phase	Infliximab	Short-term PEN (4 weeks) with general diet, non-short-term PEN	Maintenance
United States, 52 weeks [36]	Prospective pilot short- and long-term	13.06	9	M (77%) and F (23%)	Mild to moderate Crohn’s disease and requesting to seek alternative treatment besides pharmalogic	Azatiophrine, Methotrexate 6-Mercaptopurine, Budenoside, Balsalazide	Specific carbohydrate diet, none	Inclusion of remission + maintenance + follow-up after maintenance until week 52
United States, 26 months [37]	Retrospective review/retrospective observational	11	7	M (43%) and F (57%)	Children with CD and have implemented the specific carbohydrate/modified specific carbohydrate diet for >3 months	None	Modified specific carbohydrate diet, none	Maintenance
Poland, 12 weeks [38]	Prospective cohort/observational	12	48	M (56%) and F (44%)	Children with different severities of CD (from mild exacerbations to clinical remission), but increased FC level ≥ 250 µg/g	Exclusive enteral nutrition, steroid therapy, and biological treatment	CDED + PEN, none	Maintenance

^1^ Age (years) written in treatment and control group. * PCDAI = Paediatric Crohn’s Disease Activity Index; a subjective scale to measure disease activity. ** FC = faecal calprotectin; measurement of intestinal inflammation.

**Table 3:** Outcomes of the included studies.

Measured Outcome									
	Direct Evidence	Indirect Evidence							
	Urinary Lactose/Rhamnose Sugar (L/R Ratio)	Faecal Calprotectin (FCP)	BMI (Percentile/pp or kg/m^2^)	PCDAI Score	C-Reactive Protein (CRP) (mg/dL or mg/L)	Erythrocyte Sedimentation Rate (ESR) (mm/h)	Albumin (g/dL)	Simple Endoscopic Score for CD (SES-CD)	Lewis Score
Argentina [31]	x	✓	✓	✓	✓	✓	✓	x	x
United Kingdom [32]	✓	✓	✓	✓	✓	✓	×	x	x
South Korea [28]	x	x	x	✓	x	x	✓	x	x
United States [30]	x	x	✓	✓	x	✓	✓	x	✓
United States [31]	x	✓	x	x	✓	x	✓	✓	x
Poland [32]	x	✓	x	✓	✓	✓	x	x	x

✓ is reported and x is not reported.

**Table 4:** Treatment details.

Author, Country	Type of Dietary Treatment	Composition of Dietary Treatment	Total Energy and Macronutrients in Diet Treatment Group	Diet Assessment Tools (Treatment Group)	Diet Adherence and Acceptability in Treatment Group	Comparator of Treatment Group and Its Composition
Argentina [31]	CDED + PEN	Recommended: Fruits. Vegetables. White meats (fresh chicken breast or fresh fish). Simple and complex carbohydrate. Limit or eliminate: Animal fat. Meats (any type of processed, pre-cooked or smoked meats and fish, other parts of chicken, turkey, seafood, and ground beef). Gluten. Maltodextrins. Xantham gum. Emulsifiers. Sulfites.	**Phase 1 (Week 0–6):** Received 50% of solid food permitted in CDED + PEN and 50% from a polymeric liquid formula containing beta-transforming growth factor (Modulen-IBD, Nestlé Health Sciences, Switzerland). **Phase 2 (Week 7–12):** Received 75% of solid food and 25% of polymeric liquid formula.	Total energy and protein intake assessed according to age, gender, and nutritional status of included participants. Five-day dietary intake diary to assess adherence to and compliance with CDED + PEN diet.	Only one patient in CDED + PEN group had clinical relapse during 12-week period, compared with four patients in regular diet group (*p* = 0.149). One patient in CDED + PEN considered to intensify the dose of biological treatment, while there were eight patients in regular diet group (*p* = 0.005).	Regular diet: only healthy eating advised and no specific food restrictions recommended.
United Kingdom [32]	Bovine colostrum milk and continue usual diet	Bovine colostrum: produced by cows for the first few days after parturition and contains higher concentration of antimicrobial, immunomodulatory, and growth-stimulating factors than mature milk. Energy: 460 kcal/100 g and composed of 20% fat, 18% lactose and 55% protein (16% immunoglobulin).	Weeks 1–6 (blinded) and weeks 7–12 (open intervention): 20 g/day (4 rounded dessert spoons) made up of 150 mL of water, semi-skimmed or full cream milk or yoghurt. Various flavourings (e.g., vanilla or chocolate) also added to increase palatability. Can be taken 2 h after or 30 min before eating. Either in one go or split into two and stored in refrigerator for up to 3 days.	7-days daily symptom dairy at baseline, weeks 2, 6, and 12 to assess acceptability of BC.	**Acceptability** In blinded phase, 5/7 (71.4%) (BC group) and 6/7 (85.7%) (placebo milk) feeling well after drinking milk (*p* = 1.0) and feeling better at the end of the week (*p* = 0.56). While in open phase, withdrawals tended to be less common in children who already had BC in blinded phase (1/8, 12.5%) than those who switched from placebo milk (4/9, 44.4%), *p* = 0.29.	Placebo milk with continuation of usual diet: mixture of milk powder (70%) and milk protein concentrate (30% of pure milk protein concentrate).
Republic of Korea [34]	Short-term PEN and general diet	Standard oral polymeric formula. The formula consists of 14% protein, 54.5% carbohydrate, 31.5% fat, and caloric density 1.1 g/mL.	Ensure liquid in 250 mL: 8.8 g of milk with soy protein, 34.3 g of lactose-free carbohydrates, 8.8 g of corn oil, and 24 essential vitamins and minerals. Total uptake in 17 patients: 20 kcal/kg additional to normal diet for 4 weeks.	Nutritional status measurement was conducted at initial enrolment and following 1 year of treatment.	None	Non-short-term PEN and general diet: no composition mentioned.
United States [36]	Specific carbohydrate diet	(Table A3 in Appendix A)	**Formula of energy requirements** Pre-SCD: 85% of patient’s estimated caloric needs. Until week 12: 101% of patient’s estimated caloric needs. **Total energy requirements** Mean ± SE kcal/kg needs of 9 patients in week 0: 54 kcal/kg ± 4.6. Mean ± SE kcal/kg actual of 9 patients: 48 kcal/kg ± 9.9 (week 0) and 55 kcal/kg ± 7.5 (week 12).	3-day dietary records at baseline and the end of week 12. No dietary assessment collected at 52-week visit.	**Diet adherence rate:** until week 52, only 7 patients chose to continue SCD, with no data of actual consumption of SCD.	No comparator diet.
United States [37]	Modified specific carbohydrate diet	Re-exposure to the specified carbohydrate diet “illegal” foods (rice, oats, potatoes, quinoa).	No detailed explanation.	No detailed explanation.	**Upper gastrointestinal tract and ileal disease phenotype changes:** 3 patients with no ileal disease at baseline developed macroscopic ileal ulceration on mSCD. Meanwhile the rest had resolution but continued to have colonic inflammation with less activity.	No comparator diet.
Poland [38]	CDED + PEN	**No detailed list of food, but three groups:** Recommended: ensured adequate nutritional value and substrates to produce short-chain fatty acids. Neutral: added variety to the daily diet but not required to have it daily. Prohibited.	**Phase 1 (Week 0–6):** 50% from the enteral food (polymeric formula Modulen IBD, Nestlé Health with 1 kcal/1 mL or hydrolyzed protein formula for those having cow’s milk protein allergy) + 50% from the defined food list allowed in CDED + PEN. **Phase 2 (Week 7–12):** 25% from enteral food + 75% from the solid food (re-exposure of the eliminated foods in phase 1).	Total energy and protein intake assessed according to age, gender, and nutritional status of included participants. Adherence to and tolerance of diet assessed through second and third visit of dietary consultation (week 6 and week 12).	44/48 (91.67%) attended follow-up in week 6, and only 37 (77.08%) in week 12. **Reason for premature termination in 11 participants:** Lack of therapeutic efficacy (6/11). Difficulties adhering to the diet (2/11). Poor tolerance of enteral formula (1/11). Parents made decision to discontinue nutritional treatment (1/11).	No comparator diet.

**Table 5:** Results of each diet treatment.

Author, Country	Outcome or Aim of Focus of the Study	Result
Argentina [31]	(1) **Primary outcome:** Investigate the addition of CDED + PEN to ongoing medical treatment, whether it would decrease FC by a significantly greater amount than a regular diet or not. (2) **Secondary outcome:** Determine whether dietary treatment had a demonstrable clinical impact as determined by clinical relapses or needed changes in treatment and anthropometry.	**Faecal calprotectin** (1) FC level lower than 250 µg/g in 7/21 patients, with 5 patients from CDED + PEN group (*p* = 0.361). (2) In the treatment group, there were only 9 out of 11 children who had median FC level decreased by roughly 55% (750 µg/g to 339 µg/g, *p* = 0.0048). **PCDAI** (1) In the treatment group, children who had median PCDAI score > 10 (range 20–40) at the baseline reduced their score by almost 50% at week 12. (2) In the control group, children who had PCDAI score > 10 (range > 10–30) went into remission (PCDAI < 10) while the other maintained a high level (PCDAI score 30) and the other one had increased PCDAI. **BMI** (1) Increased BMI z-score in CDED + PEN group (−0.79 to −0.6, *p* = 0.7002). (2) Worsened BMI z-score in regular diet group (−0.07 to −0.5). **Laboratory markers** All markers (CRP, albumin, and ESR) not significant in CDED (*p* = 0.6835, *p* = 0.9658, and *p* = 0.8588, respectively) or regular diet (*p* = 0.1834, *p* = 0.09349, and *p* = 0.7204, respectively).
United Kingdom [32]	To assess the acceptability of bovine colostrum in children with Crohn’s disease along with identifying its effects on disease symptoms, biomarkers of intestinal integrity, inflammation and quality of life.	**wPCDAI (disease activity):** (1) No significant difference between BC and placebo from weeks 1–6. Median change/IQR from week 1–6: 10 (15-5) in BC vs. 1.25 (13.1-13.1) in placebo, *p* = 1.0. (2) BC group may have reduced disease activity from weeks 1–12: median change 15 (28.8-1.25). **Faecal calprotectin (intestinal inflammation):** During weeks 1–6, both groups showed decrease in FCP (median change −9 vs. −45.5 in BC and placebo, respectively, *p* = 0.59). **Urine sugar permeability test (mucosal integrity):** Consistent decrease in L/R ratio in BC group for both week 1 until 6 and week 7 until 12 (0.030 to 0.0046, *p* = 1.0 during first 6 weeks and 0.0021 during week 7 until 12). While for the children who started the BC at week 7, the L/R ratio established at 0.0033. **Weight changes** Weeks 1–6 (BC vs. placebo): Both groups gained a small amount of weight—a median of +1.2 kg in the BC group and +1.6 kg in the placebo group (*p* = 0.37) **Symptoms and well-being (quality of life)** Out of 10 children who completed 7-day symptoms diaries, the most common symptoms were having wind and lacking energy (IMPACT QoL score recorded >50%), both in BC and placebo group. **Laboratory markers** CRP for 12 weeks in both groups not significant (*p* = 0.21). ESR not reported.
Republic of Korea [34]	Examine the 4-week supportive short-term PEN as an adjuvant therapy after induction of remission on nutritional status and Crohn’s disease severity in children.	**Micronutrient status** Nutritional status among children with severe disease of CD (*n* = 34) improved (*p* < 0.001), except 25-OH-vitamin D after 1 year of treatment. **Differences between initial enrolment and after 1 year treatment** Body weight (10.7 ± 2.5), PCDAI (62.7 ± 16.4), zinc (45.6 ± 12.4), vitamin B12 (297.4 ± 27.1), vitamin E (7.2 ± 1.4), folate (8.4 ± 1.3), and 25-OH-vitamin D (4.3 ± 0.6) were significantly higher in SPEN group (*p* < 0.001) than in non-SPEN group (7.4 ± 4.2, 50.2 ± 17.1, 26.7 ± 21.6, 197.6 ± 42.8, 6.3 ± 3.1, 6.9 ± 3.4, and 3.8 ± 1.3, respectively). **Differences in PCDAI between enrolment and after 1 year treatment** SPEN group had greater difference (62.7 ± 16.4) than non-SPEN group (50.2 ± 17.1), *p* < 0.001. **Laboratory markers** ESR not reported.
United States [36]	Evaluate the mucosal healing in the small bowel of children with Crohn’s disease after consumption of specific carbohydrate diet (SCD).	**BMI** (1) BMI% percentile decreased across the three visits in week 0, week 12, and week 52 (30 ± 28.9, 29.3 ± 28.7, and 14.7 ± 13, respectively). (2) Weight gain at week 12 was inversely associated with PCDAI (r= −0.763, covariance −26.528). **PCDAI** (1) PCDAI declined from week 0 to week 52 (21.1 ± 5.9, 7.8 ±7.1 (*p* = 0.011), and 5.4 ± 5.5 (*p* = 0.027). **Lewis score** (1) The average of the Lewis score dropped from week 0 to 12 (2153 ± 732 to 960 ± 432, *p* = 0.012), but increased in week 52 (1046 ± 372, *p* = 0.091). **Laboratory markers** (1) ESR and albumin not reported.
United States [37]	Describe the extent of mucosal healing in children with Crohn’s disease on mSCD as their only active treatment.	**Faecal calprotectin** FCP: Checked in 5 out of 7 patients. All 5 patients had >50 μg/g, with range of 65 to 312, median 201 ± 74. **Laboratory result** (1) Albumin, haematocrit and CRP were consistently normal for 5 out 7 patients. The remaining 2 patients: 1 had albumin 3.7 (normal: >3.8 g/dL) and 1 had CRP 1.0 mg/dL (normal: <0.8 mg/dL). (2) ESR (only available to 2 patients): normal (< 20 mm/h). **SES-CD and endoscopic assessment** (1) Ileocolonic endoscopic assessment: One patient having complete ileocolonic mucosal healing. (2) Meanwhile for endoscopic severity based on SES-CD, 3 out of 7 patients had no change (2 mild, 1 moderate). Two out of 7 patients showed improvement (severe to moderate, and moderate to mild). The last 2 patients each had resolution (from severe to none) and worsened condition (moderate to severe).
Poland [38]	The effectiveness of nutritional therapy CDED + PEN towards faecal calprotectin level (non-invasive marker for mucosal inflammation) in children with active Crohn’s disease	**Faecal calprotectin** (1) The percentage and score of FC level at week 12 was higher in children who did not achieve clinical remission at baseline compared with the children who already had clinical remission at baseline: (50% vs. 27.6%) and (567 µg/g vs. 133 µg/g, *p* < 0.005), respectively. (2) Children who achieved remission (measurement at weeks 0, 6, and 12) had decreasing trends across three points: 939.5 µg/g, 587.5 µg/g, and 133 µg/g, respectively (*p* = 0.0132). (3) Children who did not achieved remission (measurement at weeks 0, 6, and 12) also had decreasing trends: 1410 µg/g, 655 µg/g, and 567 µg/g, respectively (*p* = 0.0106). **Laboratory markers** In the entire studied population (*n* = 48), significant decrease in CRP (median value from 1 mg/dL at baseline to 0.19 mg/dL in week 12, *p* = 0.0002) and ESR (from 21 mm/h at baseline to 11 mm/h at week 12, *p* = 0.0014).

## Data Availability

No new data were created or analyzed in this study. Data sharing is not applicable to this article.

## Abbreviations

The following abbreviations are used in this manuscript:

CD	Crohn’s disease
EEN	Exclusive enteral nutrition
PEN	Partial enteral nutrition
CDED	Crohn’s disease exclusion diet
ECCO/ESPGHAN	The European Crohn’s and Colitis Organisation/the European Society for Paediatric Gastroenterology, Hepatology and Nutrition
CYP	Children and young people
PCDAI	Paediatric Crohn’s Disease Activity Index
wPCDAI	Weighted Paediatric Crohn’s Disease Activity Index
FC	Faecal calprotectin
SES-CD	Simple Endoscopic Score for Crohn’s Disease
L/R ratio	Lactulose/rhamnose ratio
LS	Lewis score
BC	Bovine colostrum
SCFA	Short-chain fatty acid

## Appendix A

**Table A1:** Detailed search strategy according to databases.

**Cochrane Library**	
**# (Applied title, abstract, and keyword)**	**Query**
**S1**	“paediatric Crohns disease”
**S2**	“diet therapy”
**S3**	“Nutrition treatment”
**S4**	“disease activity”
**S5 (result)**	S1 AND S2 AND S3 AND S4
**MEDLINE via OVID**	
**# (Applied only keyword)**	**Query**
S1	Crohn Disease/
S2	Pediatrics/or Child/
S3	S1 AND S2
S4	Clinical Trial/
S5	S3 AND S4
S6	disease activity.mp.
S7 (result)	S5 AND S6
**EMBASE**	
**# (Applied only keyword)**	**Query**
S1	intestine mucosa permeability/or intestinal permeability.mp.
S2	Crohn disease/
S3	nutrition strategy.mp.
S4	diet therapy/
S5	S3 OR S4
S6	S2 AND S5
S7 (result)	S1 AND S6
**ScienceDirect**	
(refined to only research articles)	“crohn disease” AND “dietary therapy” OR “nutrition strategy” AND “intestinal barrier activity”
**Web of Science**	
**# (Applied only keyword)**	**Query**
S1	ALL = (disease activity)
S2	ALL = (“crohn disease”)
S3	ALL = (diet modification)
S4	ALL = (nutrition therapy)
S5	ALL = (diet intervention)
S6	S3 OR S4 OR S5
S7	S2 AND S6
S8 (result)	S1 AND S7

**Table A2:** Newcastle–Ottawa scale coding manual for cohort studies’ assessment.

Selection					Comparability	Outcome			Total Points
	Representativeness of the exposed cohort	Selection of the non-exposed cohort	Ascertainment of exposure	Demonstration that outcome of interest was not present at start of study	Comparability of cohorts on the basis of the design or analysis	Assessment of outcome	Was follow-up long enough for outcomes to occur?	Adequacy of follow-up of cohorts	
United States [36]	*	*	*	*	*	*	*		7
United States [37]	*	*	*	*	*	*		*	7
Poland [38]	*	*	*	*	*	*		*	7

* Represents one point on the scale.

**Table A3:** Specific carbohydrate diet instructions.

**Principle of Specific Carbohydrate Diet (Adaptation from Crohn & Colitis America Foundation Website)**
**Foods that may be eaten**
→ Fresh/frozen vegetables and legumes. → Fresh/raw/dried fruits, unsweetened juices (not from concentrate). → Navy beans, lentils, peas, split peas, most nuts (unroasted preferably nuts coming directly from shells so that nothing is added), natural peanut butter (with no sugar), lima beans, string beans. → Fresh/frozen meats, poultry, fish, eggs. → Some (natural/hard) cheeses (cheddar, Colby, Swiss, Havarti), homemade yogurt fermented >24 h (no sugar added), dry curd cottage cheese. → Honey. → Tea, coffee, mustard, vinegar, most oils.
**Foods to avoid**
→ Canned vegetables. → Canned fruits, unless packed in own juices. → All grains, including flours. → Potatoes, yams, parsnips. → Chickpeas, bean sprouts, soybeans, mung beans, fava beans, and garbanzo beans. → Seaweed and byproducts, including agar and carrageenan. → Processed, canned, breaded, smoked meats/fish. → All milk, buttermilk, commercially prepared yogurt and sour cream, heavy cream, soy/rice/potato/oat/hemp milk. → Instant tea or coffee, coffee substitutes, beer. → Canola oil, mayonnaise (due to additives), cornstarch, chocolate or carob, bouillon cubes or instant soup bases, all products made with refined sugar, sugar substitutes, Stevia, pectin, ketchup, ice cream, molasses, corn or maple syrup, baking powder, medication containing sugar, all seeds, balsamic vinegar, fructo-oligo saccharides.

**Table A4:** Proposed mechanisms by which dietary interventions may influence intestinal epithelial barrier function and mucosal immunity in Crohn’s disease.

Mechanism Domain	Mechanism Type	Proposed Primary Pathway	Tight Junction (TJ) Effects	Goblet Cell/Mucus Layer	Antimicrobial Peptide (AMP) Production	Microbiome Dependency	Key Gut Health Evidence in This Review
**Short-term PEN**	**[Mixed direct/indirect, with caution that research mostly refer to the formula within exclusive enteral nutrition (EEN)]** Formula directly reduces antigen load; microbial diversity changes are secondary.	Polymeric formula (6–8 weeks) provides bowel rest and changes intestinal microbial diversity. Polymeric formula in enteral nutrition has effect on modulating gut cytokines profile and supporting mucosal integrity [54].	Formula provision excludes dietary emulsifiers that exist in normal diet directly, thus prevent ongoing TJ membrane disruption. TGF-β1 upregulation observed with EEN/PEN → lead to claudin and occludin (tight junction proteins) stabilisation [54,55].	Bowel rest reduces inflammation, and thus might reduce mechanical and antigenic stress on goblet cells.	Not established for short-term PEN.	Low to moderate. Direct antigen-reduction effect is microbiome-independent. While microbiome shifts after EEN is a secondary consequence rather than direct effect mechanism after EEN.	Nutritional status and body weight were significantly higher in short-term PEN group (*p* < 0.001). Detailed result is in Table 5.
**Modified carbohydrate diet (mSCD)**	**[Indirect]** Works through the same gut bacteria pathway as SCD. The diet’s effect on the bowel lining remains indirect, it depends on bacterial change rather than delivering any active compound itself.	Liberalised version of SCD that permits prohibited carbohydrate in SCD (rice, grains, oats). Microbiome shift is attenuated but clinical outcomes are comparable at 12 weeks (SES-CD score 0 at week 12) [37].	Mechanism might be similar to SCD via reduced pathobiont load and SCFA-mediated bacteria to increase tight junction proteins. Evidence on direct TJ protein quantification in mSCD is limited.	Mechanism analogous to SCD with additional fibre-driven SCFA. Potential for greater goblet cell stimulation if resistant starch increases Bifidobacterium and Roseburia abundance. Data on MUC2 quantification in mSCD absent, only inferred from microbiome shifts.	Pathway inferred to parallel SCD. Permitted whole grains may enhance resistant starch fermentation → greater butyrate → potentially have the ability to preserve structural intestinal barrier. Clinical AMP data not reported for mSCD specifically.	High, but attenuated compared with strict SCD due to broader substrate. Whole grains may sustain some gut microbiota that strict SCD suppresses.	1. Out of 7 children there were still children with FC level > 250 µg/g [37] 2. Only one child had complete ileocolonic mucosal healing (SES-CD = 0) [37].
**Specific carbohydrate diet (SCD)**	**[Indirect]** SCD works through its effect on gut bacteria rather than acting directly on the bowel wall.	Restricts complex and disaccharide carbohydrates. Primary effect is through altered microbial community structure → leads to reduced dysbiosis and inflammatory metabolites [49].	Indirect: restriction of simple sugars reduces pathobiont overgrowth → leads to reducing significant loss of tight junction proteins [56]. Evidence on direct TJ protein quantification in SCD is limited.	Indirect: reduced pathobiont might lead to less MUC2 degradation (less impaired mucus barrier). Clinical evidence of mucus restoration in SCD is inferred; direct goblet cell quantification not yet reported.	Indirect: Elimination of grains, added sugar, and processed foods leads to reduced dysbiosis lowering IL-18 suppression, thus allowing Paneth cell defensin secretion [56]. No direct evidence from SCD clinical studies established toward AMP production.	High. Mechanism in carbohydrate restriction reducing pathobiont overgrowth and favouring SCFA producers [49].	Fluctuated Lewis score across 12 weeks (*p* = 0.091, Lewis score < 135 only showed in 4 children at week 12, and normal mucosa only observed in three children at week 52 [36].
**CDED + PEN**	**[Mixed direct/indirect]** Removes barrier-disrupting inputs (direct) and drives microbiome remodelling (indirect).	CDED removes dietary components that disrupt the mucosal environment (emulsifiers, excess animal fat, maltodextrin). PEN provides up to 50% of caloric needs as a polymeric formula substrate. Together they drive microbiome remodelling either toward butyrate-producing bacteria or decreased Proteobacteria.	Indirect: removal of emulsifiers as a core concept of CDED stops direct TJ membrane disruption.	Removes high-fat and emulsifier components (as the concept of CDED) that cause goblet cell endoplasmic reticulum (ER) stress and suppress MUC2 proteins [57]. [Caution: Mechanistic inference only, no direct CDED + PEN measurement.]	Mechanistically plausible, but no direct evidence in this CDED + PEN context.	Most barrier and AMP effects require a microbial shift as the primary effector step [15].	FC level lower than 250 µg/g in 5 patients received CDED + PEN (*p* = 0.361) [31]. Decreased trend in FC level both in children who achieved remission and who did not achieve remission before having CDED + PEN [38].
**Bovine colostrum (BC)**	**[Primarily direct]** Exogenous bioactive delivery; microbiome-independent.	Bioactive compounds in BC (IGF-1, TGF-β2, lactoferrin, IgG) act directly on epithelial receptors and immune cells to regulate TJ proteins, mucin secretion, and mucosal immunity.	Antimicrobial peptides in bovine colostrum (lactoferrin) have the ability to increase TJ proteins (claudin-1, occludin) [18].	The production of TJ proteins subsequently activates the secretion of mucin by goblet cells.	Antimicrobial peptides in bovine colostrum itself (lactoferrin, immunoglobulin, and lysozyme) also reduce bacterial translocation across the intestinal epithelium, preventing inflammation onset [39].	Minimal. The reshaping of the microbial composition due to the AMP was due to the secondary effect, not a required effector mechanism.	Improved intestinal permeability: Decreased lactulose/rhamnose (L/R) ratio in children receiving BC (*p* = 1.0).

Summary framework of pathway types: direct = exogenous molecular delivery; indirect = microbiome-contingent; mixed = both pathways operative.

## References

[1] Cockburn E., Kamal S., Chan A., Rao V., Liu T., Huang J.Y., Segal J.P. (2023). Crohn’s disease: An update. Clin. Med..

[2] Kellermann L., Riis L.B. (2021). A close view on histopathological changes in inflammatory bowel disease, a narrative review. Dig. Med. Res..

[3] NICE (2019). Crohn’s Disease: Management: National Institute for Health and Care Excellence. https://www.nice.org.uk/guidance/ng129.

[4] Granot M., Kopylov U., Loberman-Nachum N., Krauthammer A., Abitbol C.M., Ben-Horin S., Weiss B., Haberman Y. (2024). Differences in disease characteristics and treatment exposures between paediatric and adult-onset inflammatory bowel disease using a registry-based cohort. Aliment. Pharmacol. Ther..

[5] Atia O., Focht G., Lujan R., Ledder O., Greenfeld S., Kariv R., Dotan I., Yanai H., Gabay H., Balicer R. (2022). Perianal Crohn Disease Is More Common in Children and Is Associated with Complicated Disease Course Despite Higher Utilization of Biologics: A Population-based Study from the epidemiology group of the Israeli IBD Research Nucleus (epiIIRN). J. Pediatr. Gastroenterol. Nutr..

[6] Xu Y., Xie R., Weng Y., Fang Y., Tao S., Zhang H., Chen H., Han A., Jiang Q., Liang W. (2025). Role and mechanism of gut microbiota-host interactions in the pathogenesis of Crohn’s disease. Int. J. Color. Dis..

[7] Fetter K., Weigel M., Ott B., Fritzenwanker M., Stricker S., de Laffolie J., Hain T. (2023). The microbiome landscape in pediatric Crohn’s disease and therapeutic implications. Gut Microbes.

[8] Pastorelli L., De Salvo C., Mercado J.R., Vecchi M., Pizarro T.T. (2013). Central Role of the Gut Epithelial Barrier in the Pathogenesis of Chronic Intestinal Inflammation: Lessons Learned from Animal Models and Human Genetics. Front. Immunol..

[9] Neustaeter A., Leibovitzh H., Turpin W., Croitoru K., CCC GEM consortium (2024). Understanding Predictors of Crohn’s Disease: Determinants of Altered Barrier Function in Pre-Disease Phase of Crohn’s Disease. J. Can. Assoc. Gastroenterol..

[10] Rohr M.W., Narasimhulu C.A., Rudeski-Rohr T.A., Parthasarathy S. (2020). Negative Effects of a High-Fat Diet on Intestinal Permeability: A Review. Adv. Nutr..

[11] Chae A., Aitchison A., Day A.S., Keenan J.I. (2017). Bovine colostrum demonstrates anti-inflammatory and antibacterial activity in in vitro models of intestinal inflammation and infection. J. Funct. Foods.

[12] Ruemmele F.M., Veres G., Kolho K.L., Griffiths A., Levine A., Escher J.C., Amil Dias J., Barabino A., Braegger C.P., Bronsky J. (2014). Consensus guidelines of ECCO/ESPGHAN on the medical management of pediatric Crohn’s disease. J. Crohn’s Colitis.

[13] Tita G.V.T., Serban D.E., Chiperi L.E., Fogas C.R., Medan S.A., Tantau V.M. (2025). Exclusive enteral nutrition in Crohn’s disease pediatric patients: From clinical remission to transmural healing. Med. Pharm. Rep..

[14] Geesala R., Gongloor P., Recharla N., Shi X.Z. (2024). Mechanisms of Action of Exclusive Enteral Nutrition and Other Nutritional Therapies in Crohn’s Disease. Nutrients.

[15] Levine A., Wine E., Assa A., Sigall Boneh R., Shaoul R., Kori M., Cohen S., Peleg S., Shamaly H., On A. (2019). Crohn’s Disease Exclusion Diet Plus Partial Enteral Nutrition Induces Sustained Remission in a Randomized Controlled Trial. Gastroenterology.

[16] Häcker D., Siebert K., Smith B.J., Köhler N., Riva A., Mahapatra A., Heimes H., Nie J., Metwaly A., Hölz H. (2024). Exclusive enteral nutrition initiates individual protective microbiome changes to induce remission in pediatric Crohn’s disease. Cell Host Microbe.

[17] de Bie C., Kindermann A., Escher J. (2013). Use of exclusive enteral nutrition in paediatric Crohn’s disease in The Netherlands. J. Crohn’s Colitis.

[18] Zhao X., Xu X.X., Liu Y., Xi E.Z., An J.J., Tabys D., Liu N. (2019). The In Vitro Protective Role of Bovine Lactoferrin on Intestinal Epithelial Barrier. Molecules.

[19] Karakülah Y.S., Yalçıntaş Y.M., Bechelany M., Karav S. (2025). Clinical Applications of Bovine Colostrum in GastrointestinaI Disorders: Mechanisms, Evidence, and Therapeutic Potential. Int. J. Mol. Sci..

[20] Coskun M. (2014). Intestinal Epithelium in Inflammatory Bowel Disease. Front. Med..

[21] Marco M.L., Cunningham M., Bischoff S.C., Clarke G., Delzenne N., Lewis J.D., Meisel M., Merenstein D., O’Toole P.W., Staudacher H.M. (2026). The International Scientific Association for Probiotics and Prebiotics (ISAPP) consensus statement on the definition and scope of gut health. Nat. Rev. Gastroenterol. Hepatol..

[22] Neurath M.F., Artis D., Becker C. (2025). The intestinal barrier: A pivotal role in health, inflammation, and cancer. Lancet Gastroenterol. Hepatol..

[23] Bischoff S.C., Barbara G., Buurman W., Ockhuizen T., Schulzke J.D., Serino M., Tilg H., Watson A., Wells J.M. (2014). Intestinal permeability–a new target for disease prevention and therapy. BMC Gastroenterol..

[24] Kapel N., Ouni H., Benahmed N.A., Barbot-Trystram L. (2023). Fecal Calprotectin for the Diagnosis and Management of Inflammatory Bowel Diseases. Clin. Transl. Gastroenterol..

[25] Page M.J., McKenzie J.E., Bossuyt P.M., Boutron I., Hoffmann T.C., Mulrow C.D., Shamseer L., Tetzlaff J.M., Akl E.A., Brennan S.E. (2021). The PRISMA 2020 statement: An updated guideline for reporting systematic reviews. BMJ.

[26] Turner D., Levine A., Walters T.D., Focht G., Otley A., López V.N., Koletzko S., Baldassano R., Mack D., Hyams J. (2017). Which PCDAI Version Best Reflects Intestinal Inflammation in Pediatric Crohn Disease?. J. Pediatr. Gastroenterol. Nutr..

[27] Foster A.J., Smyth M., Lakhani A., Jung B., Brant R.F., Jacobson K. (2019). Consecutive fecal calprotectin measurements for predicting relapse in pediatric Crohn’s disease patients. World J. Gastroenterol..

[28] Tibble J.A., Bjarnason I. (2001). Non-invasive investigation of inflammatory bowel disease. World J. Gastroenterol..

[29] Rath T., Atreya R., Bodenschatz J., Uter W., Geppert C.E., Vitali F., Fischer S., Waldner M.J., Colombel J.-F., Hartmann A. (2023). Intestinal Barrier Healing Is Superior to Endoscopic and Histologic Remission for Predicting Major Adverse Outcomes in Inflammatory Bowel Disease: The Prospective ERIca Trial. Gastroenterology.

[30] Yablecovitch D., Lahat A., Neuman S., Levhar N., Avidan B., Ben-Horin S., Eliakim R., Kopylov U. (2018). The Lewis score or the capsule endoscopy Crohn’s disease activity index: Which one is better for the assessment of small bowel inflammation in established Crohn’s disease?. Ther. Adv. Gastroenterol..

[31] Arcucci M.S., Menendez L., Orsi M., Gallo J., Guzman L., Busoni V., Lifschitz C. (2024). Role of adjuvant Crohn’s disease exclusion diet plus enteral nutrition in asymptomatic pediatric Crohn’s disease having biochemical activity: A randomized, pilot study. Indian J. Gastroenterol..

[32] Allen S.J., Belnour S., Renji E., Carter B., Bray L., Allen A., Jones E., Urban B., Moule S., Wang D. (2022). Dietary Therapy to Improve Nutrition and Gut Health in Paediatric Crohn’s Disease; A Feasibility Study. Nutrients.

[33] Sterne J.A.C., Savović J., Page M.J., Elbers R.G., Blencowe N.S., Boutron I., Cates C.J., Cheng H.-Y., Corbett M.S., Eldridge S.M. (2019). RoB 2: A revised tool for assessing risk of bias in randomised trials. BMJ.

[34] Kang Y., Kim S., Kim S.Y., Koh H. (2015). Effect of Short-Term Partial Enteral Nutrition on the Treatment of Younger Patients with Severe Crohn’s Disease. Gut Liver.

[35] Sterne J.A., Hernán M.A., Reeves B.C., Savović J., Berkman N.D., Viswanathan M., Henry D., Altman D.G., Ansari M.T., Boutron I. (2016). ROBINS-I: A tool for assessing risk of bias in non-randomised studies of interventions. BMJ.

[36] Cohen S.A., Gold B.D., Oliva S., Lewis J., Stallworth A., Koch B., Eshee L., Mason D. (2014). Clinical and mucosal improvement with specific carbohydrate diet in pediatric Crohn disease. J. Pediatr. Gastroenterol. Nutr..

[37] Wahbeh G.T., Ward B.T., Lee D.Y., Giefer M.J., Suskind D.L. (2017). Lack of Mucosal Healing from Modified Specific Carbohydrate Diet in Pediatric Patients with Crohn Disease. J. Pediatr. Gastroenterol. Nutr..

[38] Matuszczyk M., Meglicka M., Wiernicka A., Jarzebicka D., Osiecki M., Kotkowicz-Szczur M., Kierkus J. (2022). Effect of the Crohn’s Disease Exclusion Diet (CDED) on the Fecal Calprotectin Level in Children with Active Crohn’s Disease. J. Clin. Med..

[39] Ulfman L.H., Leusen J.H.W., Savelkoul H.F.J., Warner J.O., van Neerven R.J.J. (2018). Effects of Bovine Immunoglobulins on Immune Function, Allergy, and Infection. Front. Nutr..

[40] Raoul P., Cintoni M., Palombaro M., Basso L., Rinninella E., Gasbarrini A., Mele M.C. (2022). Food Additives, a Key Environmental Factor in the Development of IBD through Gut Dysbiosis. Microorganisms.

[41] van Rheenen P.F., Aloi M., Assa A., Bronsky J., Escher J.C., Fagerberg U.L., Gasparetto M., Gerasimidis K., Griffiths A., Henderson P. (2021). The Medical Management of Paediatric Crohn’s Disease: An ECCO-ESPGHAN Guideline Update. J. Crohn’s Colitis.

[42] Scarallo L., Banci E., Pierattini V., Lionetti P. (2021). Crohn’s disease exclusion diet in children with Crohn’s disease: A case series. Curr. Med. Res. Opin..

[43] Verburgt C.M., Dunn K.A., Ghiboub M., Lewis J.D., Wine E., Sigall Boneh R., Gerasimidis K., Shamir R., Penny S., Pinto D.M. (2023). Successful Dietary Therapy in Paediatric Crohn’s Disease is Associated with Shifts in Bacterial Dysbiosis and Inflammatory Metabotype Towards Healthy Controls. J. Crohn’s Colitis.

[44] Zheng L., Kelly C.J., Battista K.D., Schaefer R., Lanis J.M., Alexeev E.E., Wang R.X., Onyiah J.C., Kominsky D.J., Colgan S.P. (2017). Microbial-Derived Butyrate Promotes Epithelial Barrier Function through IL-10 Receptor–Dependent Repression of Claudin-2. J. Immunol..

[45] de Jong N.S., Leach S.T., Day A.S. (2007). Polymeric formula has direct anti-inflammatory effects on enterocytes in an in vitro model of intestinal inflammation. Dig. Dis. Sci..

[46] Svolos V., Gordon H., Lomer M.C.E., Aloi M., Bancil A., Day A.S., Fitzpatrick J.A., Gerasimidis K., Gkikas K., Godny L. (2025). ECCO Consensus on Dietary Management of Inflammatory Bowel Disease. J. Crohn’s Colitis.

[47] Pigneur B., Martinez-Vinson C., Bourmaud A., Dabadie A., Duclaux-Loras R., Hugot J.P., Roman C., Dumant C., Spyckerelle C., Guinard-Samuel V. (2026). Cyclic exclusive enteral nutrition versus partial enteral nutrition to maintain long-term drug-free remission in paediatric Crohn’s disease (CD-HOPE): An open-label, endpoint-blinded, randomised controlled trial. Lancet Gastroenterol. Hepatol..

[48] Brown S.C., Whelan K., Frampton C., Wall C.L., Gearry R.B., Day A.S. (2022). Food-Related Quality of Life in Children and Adolescents with Crohn’s Disease. Inflamm. Bowel Dis..

[49] Suskind D.L., Lee D., Kim Y.M., Wahbeh G., Singh N., Braly K., Nuding M., Nicora C.D., Purvine S.O., Lipton M.S. (2020). The Specific Carbohydrate Diet and Diet Modification as Induction Therapy for Pediatric Crohn’s Disease: A Randomized Diet Controlled Trial. Nutrients.

[50] Yan J., Wang L., Gu Y., Hou H., Liu T., Ding Y., Cao H. (2022). Dietary Patterns and Gut Microbiota Changes in Inflammatory Bowel Disease: Current Insights and Future Challenges. Nutrients.

[51] Martinez-Medina M., Garcia-Gil L.J. (2014). Escherichia coli in chronic inflammatory bowel diseases: An update on adherent invasive Escherichia coli pathogenicity. World J. Gastrointest. Pathophysiol..

[52] Lewis J.D., Sandler R.S., Brotherton C., Brensinger C., Li H., Kappelman M.D., Daniel S.G., Bittinger K., Albenberg L., Valentine J.F. (2021). A Randomized Trial Comparing the Specific Carbohydrate Diet to a Mediterranean Diet in Adults with Crohn’s Disease. Gastroenterology.

[53] Britto S.L., Kellermayer R. (2020). Durable Clinical and Biochemical but Not Endoscopic Remission in Pediatric Crohn’s Disease on Specific Carbohydrate Diet Monotherapy. Ann. Clin. Lab. Sci..

[54] Pigneur B., Lepage P., Mondot S., Schmitz J., Goulet O., Doré J., Ruemmele F.M. (2018). Mucosal Healing and Bacterial Composition in Response to Enteral Nutrition vs. Steroid-based Induction Therapy—A Randomised Prospective Clinical Trial in Children with Crohn’s Disease. J. Crohn’s Colitis.

[55] Wedrychowicz A., Kowalska-Duplaga K., Jedynak-Wasowicz U., Pieczarkowski S., Sladek M., Tomasik P., Fyderek K. (2011). Serum Concentrations of VEGF and TGF-β1 During Exclusive Enteral Nutrition in IBD. J. Pediatr. Gastroenterol. Nutr..

[56] Bischoff S.C., Kaden-Volynets V., Filipe Rosa L., Guseva D., Seethaler B. (2021). Regulation of the gut barrier by carbohydrates from diet–Underlying mechanisms and possible clinical implications. Int. J. Med. Microbiol..

[57] Gulhane M., Murray L., Lourie R., Tong H., Sheng Y.H., Wang R., Kang A., Schreiber V., Wong K.Y., Magor G. (2016). High Fat Diets Induce Colonic Epithelial Cell Stress and Inflammation that is Reversed by IL-22. Sci. Rep..

